# Methylome Analysis and Epigenetic Changes Associated with Menarcheal Age

**DOI:** 10.1371/journal.pone.0079391

**Published:** 2013-11-20

**Authors:** Christiana A. Demetriou, Jia Chen, Silvia Polidoro, Karin van Veldhoven, Cyrille Cuenin, Gianluca Campanella, Kevin Brennan, Françoise Clavel-Chapelon, Laure Dossus, Marina Kvaskoff, Dagmar Drogan, Heiner Boeing, Rudolf Kaaks, Angela Risch, Dimitrios Trichopoulos, Pagona Lagiou, Giovanna Masala, Sabina Sieri, Rosario Tumino, Salvatore Panico, J. Ramón Quirós, María-José Sánchez Perez, Pilar Amiano, José María Huerta Castaño, Eva Ardanaz, Charlotte Onland-Moret, Petra Peeters, Kay-Tee Khaw, Nick Wareham, Timothy J. Key, Ruth C. Travis, Isabelle Romieu, Valentina Gallo, Marc Gunter, Zdenko Herceg, Kyriacos Kyriacou, Elio Riboli, James M. Flanagan, Paolo Vineis

**Affiliations:** 1 Department of Electron Microscopy & Molecular Pathology, The Cyprus Institute of Neurology and Genetics, Nicosia, Cyprus; 2 Department of Epidemiology and Biostatistics, School of Public Health, Imperial College London, London, United Kingdom; 3 Departments of Preventive Medicine, Paediatrics, and Oncological Sciences, Mount Sinai School of Medicine, New York, New York, United States of America; 4 Molecular and Genetic Epidemiology Unit, Human Genetics Foundation, Torino, Italy; 5 Epigenetics Group, International Agency for Research on Cancer, Lyon, France; 6 Epigenetics Unit, Department of Surgery and Cancer, Imperial College London, London, United Kingdom; 7 Institut National de la Santé et de la Recherche Médicale (INSERM), Centre for Research in Epidemiology and Population Health, Institut Gustave Roussy, Villejuif, France; 8 Nutrition, Hormones and Cancer Unit, Paris South University, Villejuif, France; 9 German Institute of Human Nutrition Potsdam-Rehbruecke, Department of Epidemiology, Nuthetal, Germany; 10 Department of Cancer Epidemiology, German Cancer Research Center [DKFZ], Heidelberg, Germany; 11 Department of Epigenomics and Cancer Risk Factors, German Cancer Research Center [DKFZ], Heidelberg, Germany; 12 Department of Epidemiology, Harvard School of Public Health, Boston, Massachusetts, United States of America; 13 Bureau of Epidemiologic Research, Academy of Athens, Athens, Greece; 14 Hellenic Health Foundation, Athens, Greece; 15 WHO Collaborating Center for Food and Nutrition Policies, Department of Hygiene, Epidemiology and Medical Statistics, University of Athens Medical School, Athens, Greece; 16 Molecular and Nutritional Epidemiology Unit, Cancer Research and Prevention Institute – ISPO, Florence, Italy; 17 Epidemiology and Prevention Unit, Fondazione IRCCS Istituto Nazionale dei Tumori, Milano, Italy; 18 Cancer Registry and Histopathology Unit, “Civile - M.P. Arezzo” Hospital, ASP Ragusa, Italy; 19 Department of Clinical and Experimental Medicine, Federico II University, Naples, Italy; 20 Public Health and Planning Directorate, Asturias, Spain; 21 Andalusian School of Public Health, Granada, Spain; 22 CIBER Epidemiología y Salud Pública (CIBERESP), Madrid, Spain; 23 Public Health Division of Gipuzkoa, BioDonostia Research Institute, Health Department of Basque Region, San Sebastian, Spain; 24 Department of Epidemiology, Murcia Regional Health Council, Murcia, Spain; 25 Navarre Public Health Institute, Pamplona, Spain; 26 Julius Center for Health Sciences and Primary Care, University Medical Center, Utrecht, the Netherlands; 27 MRC Epidemiology Unit, Cambridge Institute of Public Health, Cambridge, United Kingdom; 28 Cancer Epidemiology Unit, Nuffield Department of Clinical Medicine, University of Oxford, Oxford, United Kingdom; 29 Nutrition and Metabolism Section, International Agency for Research on Cancer, Lyon, France; 30 Centre for Primary Care and Public Health, Barts and the London School of Medicine, Queen Mary, University of London, London, United Kingdom; 31 School of Public Health, Imperial College London, London, United Kingdom; University of Nevada School of Medicine, United States of America

## Abstract

Reproductive factors have been linked to both breast cancer and DNA methylation, suggesting methylation as an important mechanism by which reproductive factors impact on disease risk. However, few studies have investigated the link between reproductive factors and DNA methylation in humans. Genome-wide methylation in peripheral blood lymphocytes of 376 healthy women from the prospective EPIC study was investigated using LUminometric Methylation Assay (LUMA). Also, methylation of 458877 CpG sites was additionally investigated in an independent group of 332 participants of the EPIC-Italy sub-cohort, using the Infinium HumanMethylation 450 BeadChip. Multivariate logistic regression and linear models were used to investigate the association between reproductive risk factors and genome wide and CpG-specific DNA methylation, respectively. Menarcheal age was inversely associated with global DNA methylation as measured with LUMA. For each yearly increase in age at menarche, the risk of having genome wide methylation below median level was increased by 32% (OR:1.32, 95%CI:1.14–1.53). When age at menarche was treated as a categorical variable, there was an inverse dose-response relationship with LUMA methylation levels (OR_12–14vs.≤11 yrs_:1.78, 95%CI:1.01–3.17 and OR_≥15vs.≤11 yrs_:4.59, 95%CI:2.04–10.33; P for trend<0.0001). However, average levels of global methylation as measured by the Illumina technology were not significantly associated with menarcheal age. In locus by locus comparative analyses, only one CpG site had significantly different methylation depending on the menarcheal age category examined, but this finding was not replicated by pyrosequencing in an independent data set. This study suggests a link between age at menarche and genome wide DNA methylation, and the difference in results between the two arrays suggests that repetitive element methylation has a role in the association. Epigenetic changes may be modulated by menarcheal age, or the association may be a mirror of other important changes in early life that have a detectable effect on both methylation levels and menarcheal age.

## Introduction

In addition to genetic changes, epigenetic changes and particularly DNA methylation can play an important role in the aetiology of chronic diseases such as cancer [1]–[5]. Gene specific promoter methylation can silence genes involved in critical cellular processes such as cell cycle regulation, DNA repair or apoptosis. At the same time, genome wide hypomethylation and in particular reduced methylation in repetitive elements such as Long Interspersed Nuclear Element-1 (LINE-1) and Alu repeats has been associated with chromosomal instability and mutations leading to chronic disease [1], [3], [4], [6]. Methylation changes are most evident in tissues such as tumour biopsies when compared to normal tissue. However, genome wide methylation changes in relation to disease have been observed in surrogate tissues such as Peripheral Blood Leukocyte (PBL) DNA. Aberrant methylation in PBLs has been previously associated with breast cancer [7], [8], colorectal adenoma [9], [10], gastric cancer [11], head and neck squamous cell carcinoma [12], and bladder cancer [13]. A recent meta-analysis of all relevant studies has shown that there is overall little evidence to support an association with cancer using surrogate assays [14]. The exception has been one study based on a large population-based case-control study, the Long Island Breast Cancer Study Project, LIBCSP, with over 2,100 peripheral blood samples, which revealed greater global and promoter specific methylation in PBLs of breast cancer cases using LUMA [15]. Although the potential influence of the disease onset on the methylome of blood DNA needs to be tested, these results suggest that methylation in PBLs DNA can serve as a biomarker for chronic diseases such as cancer; it also points to a role of aberrant methylation in carcinogenesis.

Environmental exposures influence epigenetic changes, including methylation levels, particularly *in utero* and in early life [16], [17]. In fact, genomic methylation has been shown to differ with respect to several accepted disease risk factors. These include age, race, anthropometric measures, environmental exposures and dietary factors [18]–[22]. For example, a prudent dietary pattern characterized by high intake of vegetables and fruit was shown to be associated with a lower prevalence of genomic hypomethylation (17, 19). Also, alcohol drinking and low dietary folate were found to impact on genomic DNA methylation – genome wide and gene specific [8], [19]. In addition, in a multiethnic birth cohort in New-York City [18], BMI was not found to be associated with DNA methylation, but elsewhere, in women of childbearing age, a higher BMI was associated with lower global methylation [23]. In line with the latter finding, Zhang et al. [22] showed that higher physical activity is associated with higher global methylation in a cancer free population.

Reproductive factors were also shown to impact on global DNA methylation. Terry et al. [18] showed that factors that impact on breast cancer risk, including a greater birth height, a later age at menarche, nulliparity, and a later age at first birth were associated with higher global DNA methylation levels, but these results were not replicated in other studies [8], [24]. However, the studies that investigate reproductive factors and epigenetic alterations are few [25].

In the present study we aim to investigate the impact of a number of reproductive variables on DNA methylation in PBLs of healthy individuals. The relationship was first investigated with genomic DNA methylation measurements using LUminometric Methylation Assay (LUMA) in 376 women. LUMA is a cytosine extension assay where the ratio of DNA CpG site cleavage by methylation sensitive restriction endonucleases (*Hpa*II) to the cleavage from methylation insensitive endonucleases (*Msp*I) is used to determine % global methylation. *Hpa*II cleavage occurs most frequently in CpG island promoters and repetitive elements thus methylation at these sites heavily influences the LUMA methylation estimate. Subsequently, to replicate the findings observed with LUMA, whole genome methylation patterns were obtained using Illumina 450 K in an independent group of 332 women. The Illumina 450 K array covers 485,577 CpG sites, achieving a high coverage of the entire genome, excluding repetitive elements.

## Results

### LUMA Methylation is Associated with Menarcheal Age

Demographic, anthropometric, lifestyle, and reproductive characteristics for subjects included in Stage 1 are presented in Table 1. The median LUMA genome-wide methylation in these subjects was 71.7% and the standard deviation was 5.7%. Of all the anthropometric measures and lifestyle variables examined, only age at menarche was found to significantly differ across quartiles of percent genome wide methylation (Table 2). Higher genome wide methylation was associated with a younger age at menarche (Kruskal-Wallis P-value = 0.002), and this association was significant even after Bonferroni correction for multiple testing (Table 2). Age at blood collection, height, weight, BMI, physical activity, smoking status, daily alcohol, folate consumption, age at FFTP, menopausal status, parity, breastfeeding, oral contraceptive (OC) use, hormone replacement therapy (HRT) use and highest level of education achieved did not significantly differ between subjects in methylation quartiles (Table 2).

**Table 1:** Subject demographic, anthropometric, lifestyle, and reproductive characteristics, by analysis stage.

Covariate	Metric	Stage 1	Stage 2	Stage 3
		(n = 376)*	(n = 332)*	(n = 195)
**Age**	Range	33.4–75.6	34–70	35–65
	Median	52.7	54	49
	Mean (SD±)	52.9 (9.4)	52.5 (7.1)	49.4 (7.3)
**Height**	Range	136.8–185.0	139.5–177.5	137.5–176.0
	Median	160	159.3	159
	Mean (SD)	160.1 (6.7)	159.0 (6.4)	158.7 (6.7)
**Weight**	Range	39.6–110.2	42.8–106	44–103.5
	Median	64.5	63.5	62
	Mean (SD)	66.2 (11.2)	64.4 (11.2)	63.8 (9.8)
**BMI** ±				
<25 kg/m^2^	n (%)	182 (48.4)	164 (49.4)	98 (50.3)
25–30 kg/m^2^	n (%)	141 (37.5)	118 (35.5)	7035.9)
≥30 kg/m^2^	n (%)	53 (14.1)	50 (15.1)	25 (12.8)
**Physical Activity**	n (%)	**Inactive:** 34 (9.0)		
	n (%)	**Moderately Inactive:** 85 (22.6)		
	n (%)	**Moderately Active:** 210 (55.9)		
	n (%)	**Active:** 45 (12.0)		
	n (%)	**Missing:** 2 (0.5)		
	Range		1–5	0.5–30
	Median		3	8.5
	Mean (SD)		2.6 (0.8)	10.0 (6.9)
**Smoking Status**				
Current Smoker	n (%)	79 (21.1)	69 (20.9)	35 (17.9)
Former Smoker	n (%)	65 (17.4)	66 (20.0)	48 (24.6)
Never	n (%)	230 (61.5)	195 (59.1)	112 (57.5)
**Daily alcohol consumption (g/day)**	Range	0–51.2	0–88.7	0–62.6
	Median	3.5	1.9	3.3
	Mean (SD)	6.5 (8.4)	8.7 (13.1)	9.6 (13.0)
**Daily folate consumption (µg/day)**	Range	90.4–1113.0	45.3–586.2	52.6–644.8
	Median	268.5	236.1	264.1
	Mean (SD)	291.5 (107.9)	247.3 (82.0)	276.9 (95.4)
**Age at Menarche**				
≤11 yrs	n (%)	72 (19.4)	62 (18.8)	47 (24.1)
12–14 yrs	n (%)	242 (65.2)	233 (70.9)	134 (68.7)
≥15 yrs	n (%)	57 (15.4)	34 (10.3)	14 (7.2)
**Age at FFTP** ±				
<25 yrs	n (%)	147 (45.6)	118 (35.5)	65 (41.9)
25–30 yrs	n (%)	131 (40.7)	118 (35.5)	69 (44.5)
>30 yrs	n (%)	44 (13.7)	96 (29.0)	21 (13.6)
**Parous**				
No	n (%)	45 (12.0)	29 (8.8)	40 (20.5)
Yes	n (%)	330 (88.0)	301 (91.2)	155 (79.5)
**Breastfeeding**				
No	n (%)	109 (30.0)	108 (32.7)	75 (48.4)
Yes	n (%)	255 (70.0)	222 (67.3)	80 (51.6)
**Menopausal Status**				
Premenopausal	n (%)	175 (46.5)	155 (46.7)	90 (46.2)
Postmenopausal	n (%)	201 (53.5)	177 (53.3)	105 (53.8)
**HRT** ± **use**				
Ever	n (%)	29 (7.8)	13 (3.9)	34 (17.4)
Never	n (%)	343 (92.2)	317 (96.1)	158 (81.0)
**OC** ± **use**				
Ever	n (%)	176 (46.9)	131 (39.7)	94 (48.2)
Never	n (%)	199 (53.1)	199 (60.3)	101 (51.8)

* Failure of category counts to add up to this value denotes missing values.

± SD: Standard Deviation, BMI: Body Mass Index, FFTP: First Full Term Pregnancy, HRT: Hormone Replacement Therapy, OC: Oral Contraceptive.

**Table 2:** Anthropometric and lifestyle variables in healthy controls with respect to LUMA genome wide methylation quartiles (Stage 1).

Variable		Methylation Quartile: Cut-offs				
		1: 23.0–68.4%	2: 68.5–71.7%	3: 71.8–74.0%	4: 74.1–80.0%	p-value^a^
	Units	(n = 103)	(n = 91)	(n = 93)	(n = 89)	
**Age at blood collection**	Mean ± SD	52.9±8.5	52.2±8.5	53.1±10.0	53.6±10.7	0.863
**Height**	Mean ± SD	159.0±6.1	160.7±6.4	160.8±6.7	160.0±7.5	0.424
**Weight**	Mean ± SD	66.0±12.3	66.5±10.5	64.8±11.3	67.7±10.4	0.316
**BMI** ±	Mean ± SD	26.2±5.1	25.8±4.1	25.1±4.4	26.5±4.4	0.112
**Physical Activity**						
Inactive	N, (%)	4 (3.9)	9 (9.9)	11 (11.8)	10 (11.2)	
Moderately Inactive	N, (%)	29 (28.2)	22 (24.2)	16 (17.2)	18 (20.3)	
Moderately Active	N, (%)	60 (58.3)	45 (49.4)	54 (58.1)	51 (57.3)	
Active	N, (%)	9 (8.7)	15 (16.5)	11 (11.8)	10 (11.2)	
Missing	N, (%)	1 (0.9)	0 (0.0)	1 (1.1)	0 (0.0)	0.404
**Smoking Status**						
Current Smoker	N, (%)	58 (56.3)	59 (64.8)	49 (52.7)	47 (52.9)	
Former Smoker	N, (%)	23 (22.3)	12 (13.2)	20 (21.5)	22 (24.7)	
Never	N, (%)	22 (21.4)	20 (22.0)	23 (24.7)	19 (21.3)	
Unknown	N, (%)	0 (0.0)	0 (0.0)	1 (1.1)	1 (1.1)	0.596
**Alcohol consumption –lifetime average (g/day)**	Mean ± SD	7.0±8.6	6.9±9.7	5.4±6.4	6.7±8.6	0.822
**Dietary folate intake (g/day)**	Mean ± SD	294.6±121.9	276.9±96.7	293.1±104.2	301.0±105.3	0.386
**Age at menarche**	Mean ± SD	13.2±1.7	13.3±1.7	12.5±1.4	12.8±1.7	**0.002** *
**Age at FFTP** ±	Mean ± SD	25.3±3.5	26.2±3.9	24.8±4.0	25.0±3.7	0.132
**Menopausal Status**						
Pre	N, (%)	42 (40.8)	47 (51.6)	46 (49.5)	40 (44.9)	
Post	N, (%)	60 (58.3)	43 (47.3)	45 (48.4)	49 (55.1)	
Surgical Post	N, (%)	1 (0.9)	1 (1.1)	2 (2.1)	0 (0.0)	0.545
**Parous**						
No	N, (%)	12 (11.7)	7 (7.7)	13 (14.0)	19 (21.3)	
Yes	N, (%)	90 (87.4)	84 (92.3)	79 (84.9)	70 (78.7)	
Unknown	N, (%)	1 (0.9)	0 (0.0)	1 (1.1)	0 (0.0)	0.075
**Breastfeeding**						
No	N, (%)	30 (29.1)	19 (20.9)	26 (28.0)	34 (38.2)	
Yes	N, (%)	70 (68.0)	70 (76.9)	62 (66.7)	53 (59.6)	
Unknown	N, (%)	3 (2.9)	2 (2.2)	5 (5.3)	2 (2.2)	0.086
**OC** ± **use**						
No	N, (%)	50 (48.5)	53 (58.2)	47 (50.5)	49 (55.1)	
Yes	N, (%)	53 (51.5)	38 (41.8)	46 (49.5)	39 (43.8)	
Unknown	N, (%)	0 (0.0)	0 (0.0)	0 (0.0)	1 (1.1)	0.512
**HRT** ± **use**						
No	N, (%)	93 (90.3)	84 (92.3)	81 (87.1)	85 (95.5)	
Yes	N, (%)	3 (2.9)	6 (6.6)	10 (10.7)	4 (4.5)	
Unknown	N, (%)	7 (6.8)	1 (1.1)	2 (2.2)	0 (0.0)	0.399
**Highest Education**						
None	N, (%)	10 (9.6)	6 (6.6)	7 (7.4)	6 (6.8)	
Primary	N, (%)	45 (43.7)	41 (45.1)	36 (38.7)	35 (39.3)	
Technical/Professional	N, (%)	15 (14.6)	9 (9.9)	18 (19.4)	20 (22.5)	
Secondary	N, (%)	12 (11.7)	21 (23.1)	17 (18.3)	17 (19.1)	
University	N, (%)	12 (11.7)	13 (14.3)	14 (15.1)	10 (11.2)	
Unspecified	N, (%)	9 (8.7)	1 (1.0)	1 (1.1)	1 (1.1)	0.264

a For continuous variables, P-value was derived from Kruskal-Wallis test. For categorical variables, P-value was derived from a chi square test, with the exclusion of “Unknown” categories due to their small cell counts. Both reflect the association between quartiles of methylation and the investigated variables.

* Significant at the Bonferroni-corrected significance cut off (P = 0.003) for multiple comparisons.

± BMI: Body Mass Index, FFTP: First Full Term Pregnancy, HRT: Hormone Replacement Therapy, OC: Oral Contraceptive.

The association between age at menarche and methylation was further examined using logistic regression to adjust for potential confounders. When median methylation was used as a cut off, 194 subjects had methylation below median levels and 182 had methylation levels above median. Using these two classes, logistic regression showed that age at menarche was significantly associated with class occupancy. As shown in Table 3, for every yearly increase in age at menarche, the risk of having below median methylation was increased by 32% (OR: 1.32, 95%CI: 1.14–1.53). When age at menarche was treated as a categorical variable, there was an inverse dose-response relationship with methylation levels: for the age category 12–14 compared to ≤11 years the OR was 1.78 (95% CI: 1.01–3.17), and for the age category ≥15 compared to ≤11 years the OR was 4.59 (95% CI: 2.04–10.33) (P for trend<0.0001). These significant associations persisted even after adjustment for relevant confounders: centre, plate number, age at blood collection, height, weight, total physical activity, smoking status, daily alcohol consumption, and daily folate consumption.

**Table 3:** Logistic Regression for percent genome wide methylation (LUMA levels below vs. above median) by age at menarche as a categorical variable and other relevant confounders.

Variable	MethylationMedian ± SD	Adjusted OR^a^	95% Confidence Interval	P-value
**Center**	NA	0.99	0.97–1.02	0.518
**Plate number**	NA	0.94	0.80–1.10	0.400
**Age at blood collection** (continuous)	NA	0.98	0.96–1.00	0.254
**Height** (continuous in cm)	NA	0.98	0.95–1.01	0.354
**Weight** (continuous in kg)	NA	1.01	0.98–1.03	0.453
**Total physical activity index – sex specific**(continuous activity categories)	NA	1.03	0.78–1.37	0.813
**Smoking status**				
** Never**	71.62±6.25	1.00		
** Past smoker**	72.18±4.51	0.85	0.46–1.55	0.592
** Current smoker**	70.73±5.17	0.98	0.56–1.72	0.954
**Daily alcohol intake** (continuous in g/day)	NA	1.01	0.99–1.04	0.354
**Daily folate intake** (continuous in µg/day)	NA	1.00	0.99–1.00	0.863
**Age at menarche** (continuous in years)	NA	**1.32**	1.14–1.53	**<0.0001** *
**Age at menarche** (categorical)				
** ≤11 years old**	72.59±4.49	**1.00**		
** 12–14 years old**	71.62±5.93	**1.78**	1.01–3.17	**0.048** *
** ≥15 years old**	70.12±6.33	**4.59**	2.04–10.33	**<0.0001** *
** P for Trend**			**<0.0001** *	

a Each OR is adjusted for all other variables in the table.

* Significant at the 0.05 level.

### Illumina 450 k Methylome Analysis Identifies an Epi-allele Associated with Menarcheal Age

**Figure 1:**
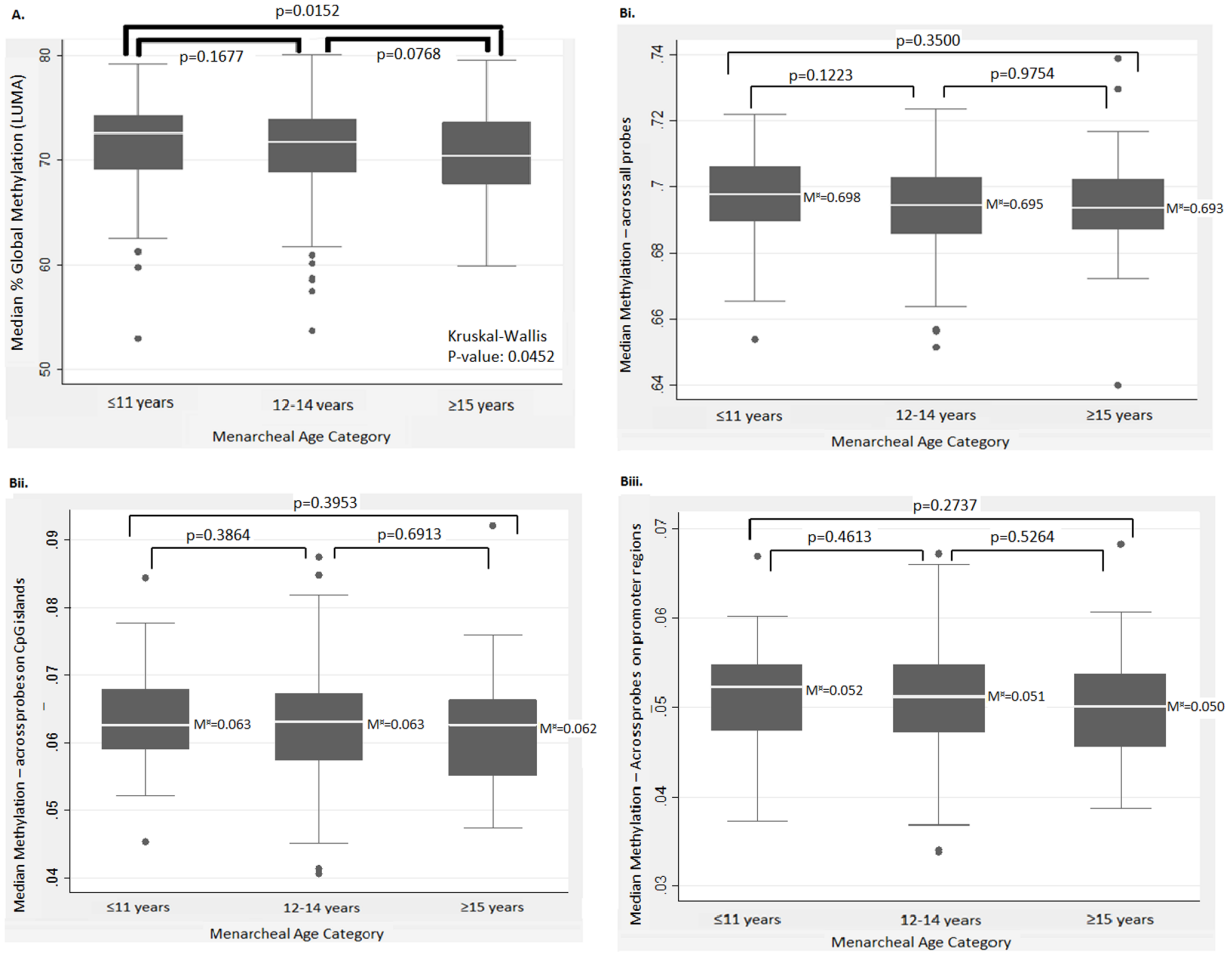
Boxplots of median genome-wide methylation between the three menarcheal age categories. A: Median % global methylation as measured with LUMA in Stage 1. Bi. Genome-wide methylation across all probes (averaged per individual). Bii. Genome-wide methylation across probes on CpG islands (averaged per individual). Biii. Genome-wide methylation across probes on promoter regions (averaged per individual). M^¤^ = Median methylation value. p = p value from Wilcoxon rank-sum test comparisons.

In the second population group, 329 subjects (out of 332) had available information on age at menarche (Table 1). In contrast to LUMA global methylation, the median genome-wide methylation level using the 450 k ILLUMINA assay did not significantly differ between menarcheal age groups. Similarly, CpG island methylation and promoter methylation were not significantly different between subjects in different menarcheal age categories. However, there was a trend towards decreasing methylation with increasing age at menarche, consistent with the LUMA results (Figure 1).

**Figure 2:**
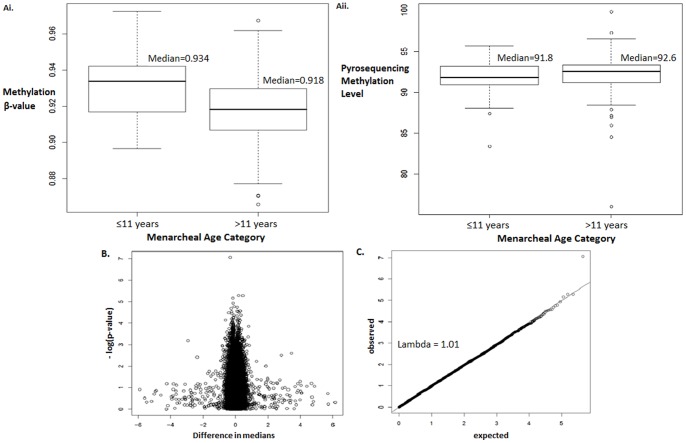
Analysis of SMAD6 cg01339004 probe methylation. Ai: Boxplot of β-value methylation of cg01339004 probe as measured with Illumina 450 k beadchip in Stage 2. Aii: Boxplot of methylation level of cg01339004 probe as measured with bisulphite pyrosequencing in Stage 3. B: Volcano plot: Difference in median methylation between the two menarcheal age groups (>11 (n = 268) vs. ≤11 years, (n = 62), against the –log(P-Value) of a linear regression analysis with methylation as a continuous outcome (M-values) and age at menarche (>11 vs. ≤11 years) as a categorical exposure, adjusting for age, case-control status, and chip position. C. Q-Q plot on P-values from a linear regression analysis with methylation as a continuous outcome (M-values) and age at menarche (>11 vs. ≤11 years) as a categorical exposure, adjusting for age, case-control status, and chip position.

When adjusting for case-control status, age, and position on the chip in a linear regression model with methylation M-values as a continuous outcome, and age at menarche as a categorical variable (>11 yrs vs. ≤11 yrs), age at menarche was significantly associated with methylation in a single CpG site (cg01339004), located on the body of the SMAD6 gene (p<1.00×10^−7^, genome-wide level significance) (Table 4, Figure 2). When only those subjects that remained healthy for at least 5 years following recruitment and blood collection were analysed, the same CpG site was found to be significantly associated with age at menarche (p = 6.71×10^−8^).

**Table 4:** Significant CpG sites in a linear regression model.

TargetID	P-value∝	Q-value≠	RegressionCoefficient≈	Chromosomenumber	Gene	CpG Position Relative to Gene	CpG Island’s Name
cg01339004	8.83E-08	0.0392	−0.2765	15	SMAD6	Body	NA

Methylation treated as a continuous outcome (M-values: PBC and COMBAT on chip) and menarcheal age category (>11 vs. ≤11 years) treated as a categorical exposure. Adjusting for age at blood collection, case-control status, and position on the chip.

Analysis of all subjects or of only the 240 subjects that remained healthy for at least 5 years following recruitment yielded the same results.

∝ P value from a liner regression model where methylation is treated as a continuous outcome (M-values: PBC and COMBAT on chip) and the effect of age at menarche as a categorical variable (>11 vs. ≤11 years), adjusted for age, case-control status, and chip position.

≠ Q value: False Discovery Rate (FDR) corrected P-value.

≈ The regression coefficient for each probe; change in methylation for having an age at menarche >11 years vs. ≤11 years.

However, using bisulphite Pyrosequencing for the SMAD6 cg01339004 locus, we were unable to replicate this finding in an independent sample set using a generalized linear model while adjusting for the same confounders (n = 185, p = 0.07). Wilcoxon rank sum non-parametric test also did not reveal significantly differential methylation between the two age at menarche categories (p = 0.082) measured using bisuphite pyrosequencing (Figure 2).

## Discussion

In this study, age at menarche was negatively associated with LUMA genome wide methylation in a statistically significant manner. The association of genome wide methylation with menarcheal age was the only strong and consistent association we found and remained unaltered after adjustment for relevant confounders. Previous study results on age at menarche and methylation were conflicting. Terry et al. [18] found that a later age at menarche was associated with higher genomic global methylation later on in adulthood, but DNA methylation was only assessed in 92 individuals and the authors used a different technique for measuring global methylation ([^3^H]-methyl acceptance assay). On the other hand, Choi et al. [8] did not demonstrate a statistically significant association between menarcheal age and global DNA methylation using LINE1 methylation as a surrogate for global methylation.

The negative association between LUMA methylation and later age at menarche is counter-intuitive because (a) a later age at menarche is known to protect from breast cancer, and (b) lower global methylation is expected to increase genome instability and thus increase cancer risk [8], [26]. However, our observation is consistent with the findings in the LIBCSP study, where breast cancer was associated with increased genome wide methylation as measured with LUMA [15]. This apparent paradox could be explained by LUMA’s characteristics, i.e. broad coverage in CpG dense regions, such as promoters, and decreased coverage in the remaining genome [27]. Another potential explanation is that aberrant methylation associated with age at menarche is unrelated to the methylation changes relevant to breast cancer, or that the association with age at menarche is in fact confounded by other determinants of methylation levels.

Given the conflicting reports in the literature [8], [15], [18], we aimed to replicate, in a dataset with whole genome methylation data, the association between age at menarche and DNA methylation that we observed with the LUMA technology. This was done by using the robust Illumina technology. This approach also enabled the identification of specific genes that might be involved in the mechanistic pathways linking menarcheal age with disease. In contrast to the findings of LUMA, genome wide methylation in this second dataset did not significantly differ between subjects in different menarcheal age groups. However, there was a non-significant trend towards decreasing methylation with increasing age at menarche, consistent with the LUMA findings (Figure 1). The lack of association in this dataset could be caused by differences in coverage between the two assays used. LUMA assesses methylation of a specific restriction enzyme site (HpaII, CCGG), which occurs most frequently in CpG island promoters – also covered by the 450 K array – but also in repetitive elements. However, the Infinium HumanMethylation 450 BeadChip, due to its probe design, does not interrogate repetitive elements which are found to be differentially methylated in many cases of neoplasia [28], [29]. For example, satellite and SINE repeats were found to be enriched with hypomethylated Differentially Methylated Regions (DMRs) whereas LINE was enriched with hypermethylated DMRs in malignant peripheral nerve sheath tumours compared to normal Schwann cells [30]. If age at menarche is related to methylation patterns in these repetitive elements, the hypomethylation would not have been evident in the 450 K chip but it would have been detected in the LUMA assay. This suggests that the LUMA based association is being driven largely by methylation differences in repetitive elements, where age at menarche could have a greater effect.

In the locus by locus analysis, methylation of a single CpG site was shown to be associated with age at menarche. However, in an independent sample set, this finding was not replicated. Given the multiple comparisons in the locus by locus analyses in the Illumina dataset, one cannot rule out the possibility that this finding is the result of chance, and given that the independent sample set did not replicate this finding using an alternative method, we conclude that it is likely to be a false positive association. However, further validation in further independent data sets, with a greater sample size may increase the power sufficiently to detect possible associations between methylation of individual loci and age at menarche.

The mechanistic link which could explain the association between menarcheal age and genome-wide DNA methylation, but not in individual CpG loci is yet to be determined. However, endogenous oestrogen exposure is a strong candidate for epigenetic changes since an earlier age at menarche exposes a woman to a greater cumulative amount of endogenous oestrogens over her lifetime, and there have been reports which show that oestrogen impacts on DNA methylation. More specifically it was shown that oestrogen receptor (ER) positive breast tumour tissues have differential methylation at several CpG loci compared to ER negative tumours [24], [31] and oestrogen induced breast tumours have differential DNA methylation patterns in ACI rat mammary gland tissue [32]. Further investigation into the role of oestrogen on repetitive element methylation is, therefore, warranted.

It is also possible that age at menarche is an indirect indicator of other macroscopic changes that may impact on DNA methylation. It is widely accepted that development is plastic and the sensitivity of the epigenetic system to environmental factors is heightened during periods of developmental plasticity such as childhood, adolescence and puberty [17]. Epigenetic modifications in response to environmental exposures at these critical periods are often subtle initially and even though they do not lead to phenotypic changes at the time of exposure, they may lead to increased risk of dysfunction and disease later on in life [17]. Trends in the past decades show a rapid shift towards an earlier age at menarche and this is more pronounced in developed countries [33]. This trend is too steep to be attributed to genetic changes. Instead, environmental exposures at the periods of developmental plasticity are likely to be the cause of the dramatic decrease in age at menarche. For example, childhood obesity disrupts the hormonal milieu leading to an increase in adipocyte secreted leptin, or in adrenal secreted androgens, all of which impact on menarcheal onset [34]. Pre or neo-natal nutrition as well as early life exposure to endocrine disrupting chemicals (EDCs) can also lead to hormonal imbalances impacting on age at menarche [17]. Given that these exposures occur at the periods when the epigenetic signature is more plastic, they might also lead to aberrant DNA methylation changes which will be inherited during cell divisions and be detectable years later. Therefore, the aberrant DNA methylation pattern observed in adulthood might not be related to menarcheal age per se but to an early life environmental exposure, like diet, that impacts both on age at menarche and on DNA methylation.

This study suggests an association between age at menarche and DNA methylation. All samples in this study were collected prior to the onset of disease, and the changes observed were present in the blood of individuals when they were still healthy, at least five years prior to their diagnosis, limiting the potential influence of the presence of cancer (reverse causality) on the methylome of blood DNA. In addition, the sample sizes examined –376 subjects for LUMA and 332 subjects for Illumina 450 K Methylation are fairly large datasets, allowing for sufficient power to detect significant methylation changes if present. However, one important limitation of our study was the lack of information on other early life exposures, therefore it was impossible to investigate whether such exposures confound the observed association between age at menarche and DNA methylation. Thus, this hypothesis needs to be further investigated in birth cohorts.

Overall, our results suggest that DNA methylation changes, particularly in repetitive elements, may be associated with menarcheal age. However, it is also possible that some important changes taking place in early life and which are associated with age at menarche – in particular nutrition – have a detectable effect on methylation levels.

## Materials and Methods

### Stage 1: Genome Wide Methylation with LUminometric Methylation Assay (LUMA)

#### Study participants

All participants signed an informed consent and the study protocol was approved by the Ethics Committee of the International Agency for Research on Cancer.

Epidemiologic data and blood samples collected from the European Prospective Investigation into Cancer and Nutrition (EPIC) were used. EPIC is an ongoing study designed to investigate diet, nutrition, lifestyle and environmental factors with respect to cancer incidence. The cohort consists of 519,978 participants from 23 centres in 10 European countries - Denmark, France, Germany, Greece, Italy, the Netherlands, Norway, Spain, Sweden and the United Kingdom. Information on lifestyle, diet, anthropometric measures and environmental exposures were collected using questionnaires at recruitment and were standardized across the different participating centres. Blood was also collected from the majority of subjects at recruitment [35]. For the LUMA investigation, 600 individuals – half breast cancer cases and half controls – from the EPIC cohort were chosen. Of these, 77 subjects were initially excluded: 1 subject was a duplicate, there was not enough DNA for 24 subjects, and 52 samples produced no or a weak signal. Of the remaining 523 subjects with reliable measurements, we investigated 376 women in this specific study, who remained free of cancer for at least 5 years following blood collection.

#### Genome wide DNA methylation

LUMA was used to quantify genome wide methylation levels [27], [36] in PBLs in the blood of subjects, collected at recruitment. Genomic DNA was extracted using standard protocols. LUMA gives a measure of % global methylation using the ratio of DNA cleavage by methylation sensitive (HpaII) and methylation insensitive (MspI) restriction enzymes. In LUMA, polymerase extension assay by Pyrosequencing is employed to determine cleavage. The LUMA method was validated using DNA controls of known DNA methylation status [37]. In the assay, 5-Aza-dC treated and CpG methylated Jurkat genomic DNA (New England Biolabs, Ipswich, MA) were used as methylated and unmethylated control samples. Genome wide methylation is expressed as a percentage obtained from the equation [37]:
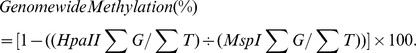



#### Statistical analyses

We first compared the distribution of a number of anthropometric measures, reproductive factors, and lifestyle characteristics such as age at blood collection, height, weight, parity, age at first full term pregnancy, breastfeeding and hormone use across quartiles of percent global methylation. The quartile cut offs were the 25^th^, 50^th^, and 75^th^ percentile methylation values in controls. For continuous variables, the non-parametric equivalent of one-way analysis of variance (ANOVA), the Kruskal-Wallis test, was used as genome wide methylation was not normally distributed. For categorical variables, chi-square test was used.

The reproductive variables that were statistically differentially distributed between methylation quartiles were investigated further. The resulting profile of genome wide methylation distribution was skewed and several transformations failed to normalize it. In addition, various GLM models investigated failed to adequately describe the outcome distribution. As a result, the methylation outcome was dichotomized – above and below median methylation – and unconditional logistic regression was used to evaluate the association between exposure variables and DNA methylation, the latter being the dependent variable. All significant variables were included both as continuous and categorical when relevant (e.g. age at menarche ≤11 y, 12–14 y, ≥15 y). Based on the available literature, the logistic regression model was fully adjusted for centre, plate number, age at blood collection, height, weight, total physical activity, smoking status, daily alcohol consumption, and daily folate consumption. All confounders were entered into the model as continuous variables with the exception of smoking status which was treated as categorical – past, never, present.

All analyses were performed using STATA (Release 11; College Station, TX: StataCorp LP).

### Stage 2: Locus-by-locus Analysis with Illumina 450 K to Replicate the Findings of LUMA Genome Wide Methylation Analysis

#### Study participants

All participants signed an informed consent and the study protocol was approved by the ethics committee of the Human Genetics Foundation (HuGeF).

The EPIC Italy sub-cohort consists of 32,578 female subjects recruited from 5 different centers – Varese, Turin, Florence, Naples, and Ragusa. From this subcohort, 166 breast cancer cases and 166 controls, matched on date of birth (±5 years), seasonality of blood draw, and date of recruitment were selected. However, since for this investigation case/control status is not the outcome and since at the time of blood collection all individuals were healthy, all 332 blood samples were treated as healthy blood. Nevertheless, given the long latency period of neoplasia, analyses were carried out on all subjects with age at menarche information (n = 329) as well as on only the subjects that remained healthy at least 5 years following recruitment and blood collection (n = 240).

#### Illumina 450 K methylation

DNA was extracted from buffy coats or blood cell fractions using the QIAsymphony DNA Midi Kit (Qiagen, Crawley, UK). 500 ng of DNA was bisulphite-converted with the EZ-96 DNA Methylation-Gold™ Kit, used according to the manufacturer’s protocol (Zymo Research, Orange, CA, USA). Next, the 450 K DNA methylation array by Illumina (Infinium HumanMethylation 450 BeadChip) was performed on 4 µl of bisulphite-converted DNA, following the Illumina Infinium HD Methylation protocol. This array includes 485,577 cytosine positions of the human genome (482,421 CpG sites (99.4%), 3091 non-CpG sites and 65 random SNPs; hereafter the term CpG will be used to refer to all of these, unless otherwise specified). Briefly, a whole genome amplification step was followed by enzymatic end-point fragmentation and hybridization to HumanMethylation 450 BeadChips at 48°C for 17 h, followed by single nucleotide extension. The incorporated nucleotides were labelled with biotin (ddCTP and ddGTP) and 2,4-dinitrophenol (DNP) (ddATP and ddTTP). After the extension step and staining, the BeadChip was washed and scanned using the Illumina HiScan SQ scanner. The intensities of the images were extracted using the GenomeStudio (v.2011.1) Methylation module (1.9.0) software, which normalizes within-sample data using different internal controls that are present on the HumanMethylation 450 BeadChip and internal background probes. The Infinium HumanMethylation 450 BeadChip data, for subjects with age at menarche information, were made available on the data repository Gene Expression Omnibus (GEO), with accession number GSE51057.

#### Statistical analyses

Methylated and unmethylated intensities for each probe were provided by GenomeStudio software. In addition to the methylation value for each probe, corresponding detection p-values were also provided by GenomeStudio software (Illumina). The detection values indicate the confidence that can be placed on a β-value reading. As a first step, all readings with a p-value>0.05 were considered as non-detected so as to not influence downstream pre-processing and analyses.

Background noise correction was then performed as background fluorescence can contribute an additive error to each signal intensity leading to a reduced dynamic range for the methylation reading. Given that signal intensities can be red or green, and given the technical variation in fluorescent signal depending on the intensity colour, dye bias also had to be taken into account using the method described by Triche et al. [38]. The analysis of other classical quality control measures (such as staining, extension, hybridization, or bisulphite conversion) provided by GenomeStudio did not reveal any major quality issues.

The methylated and unmethylated intensities provided by GenomeStudio were used to calculate methylation β-values based on the equation:
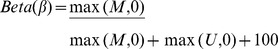
where M is the intensity of the methylated signal and U the intensity of the unmethylated signal at each probe.

Beta values were later peak based corrected (PBC) as suggested by Dedeurwaerder et al. [39] in order to correct for the bias arising from the two different probe designs on the array. In order to correct for batch effects, COMBAT was then used [40], [41]. Lastly, missing data were imputed using KNN (k-nearest neighbours) method once, implemented in knn.impute function from R-CRAN.

In order to replicate the results observed with LUMA, methylation beta values across all probes were averaged per individual to derive a measure of genome-wide methylation per subject. Similarly, probes in CpG islands and promoter regions were averaged per subject. Wilcoxon-rank sum tests were performed to examine whether genome-wide, CpG island and promoter methylation was significantly different between subjects in the three menarcheal age groups examined with LUMA (≤11 y, 12–14 y, ≥15 y).

In addition, locus by locus analysis of methylation was performed using a linear regression model. Quantile normalization was performed prior to regression using the R package “preprocessCore” from Bioconductor. In order to satisfy the normality assumptions of a linear regression model, beta methylation values were converted to M-values as described in [42] and entered into the model as a continuous outcome. M-values were also peak based corrected and COMBAT adjusted for chip number to correct for batch effects. Only age at menarche, the only significant reproductive variable in LUMA analysis, was investigated here. Age at menarche was treated as a categorical outcome (≤11 yrs vs. >11 yrs). This categorization was chosen since in the LUMA data, both menarcheal age categories above 11 yrs old were significantly associated with methylation when compared to a menarcheal age of ≤11 years.

Q-values, measuring the maximum False Discovery Rate (FDR) from the Benjamini and Hochberg method were derived for each probe analysis, and overall Type I error was controlled for by conservatively applying Bonferroni multiple testing correction. The per-test significance cut-off value was set to 1.00×10^−7^. The analysis was performed first on all 329 subjects with age at menarche information and then repeated only on the 240 subjects that remained healthy for at least five years following recruitment. The linear model was adjusted for age, case-control status and chip position. All confounders were entered into the model as continuous variables with the exception of chip position.

### Stage 3: Single Locus (SMAD6, cg01339004) Pyrosequencing Analysis to Replicate the Findings of Locus by locus Illumina 450 K Analysis

#### Study participants

The participants used in this stage were also subjects of the EPIC Italy sub-cohort. One hundred ninety-five women, with available information on age at menarche, were selected for bisulphite pyrosequencing based on sample availability from other studies. Eight of these subjects overlapped with the subjects used in Stage 2.

Bisulphite Pyrosequencing was used to quantify CpG specific methylation at the SMAD6 locus in these individuals using standard protocols [43]. The primers used for cg01339004 were Forward ([BIOTIN]–TGGTATAGTAGTGGTTTGGTATAAGAT), Reverse (TACCACCCACCCATTCACTCTATAA) and Sequencing Primer (TCTATAAATAAACAAACTAAAACC).

#### Statistical analyses

Out of the 195 samples analysed, for 10 samples the pyrosequencing results did not pass quality check. Wilcoxon rank sum non-parametric test was performed to examine whether SMAD6 cg01339004 methylation was different between age at menarche categories (≤11 vs. >11 years). In addition, a generalized linear regression model with SMAD6 cg01339004 methylation as a continuous outcome and with age at menarche as a categorical variable (≤11 vs. >11 years) was run to correct for confounding variables – age at blood collection and case-control status – as in the case of the Illumina 450 K analysis.
